# Rab5 Isoforms Orchestrate a “Division of Labor” in the Endocytic Network; Rab5C Modulates Rac-Mediated Cell Motility

**DOI:** 10.1371/journal.pone.0090384

**Published:** 2014-02-28

**Authors:** Pin-I Chen, Kristine Schauer, Chen Kong, Andrew R. Harding, Bruno Goud, Philip D. Stahl

**Affiliations:** 1 Department of Cell Biology and Physiology, Washington University School of Medicine, St. Louis, Missouri, United States of America; 2 Molecular Mechanisms of Intracellular Transport, Institut Curie, Paris, France; King's College London, United Kingdom

## Abstract

Rab5, the prototypical Rab GTPase and master regulator of the endocytic pathway, is encoded as three differentially expressed isoforms, Rab5A, Rab5B and Rab5C. Here, we examined the differential effects of Rab5 isoform silencing on cell motility and report that Rab5C, but neither Rab5A nor Rab5B, is selectively associated with the growth factor-activation of Rac1 and with enhanced cell motility. Initial observations revealed that silencing of Rab5C expression, but neither Rab5A nor Rab5C, led to spindle-shaped cells that displayed reduced formation of membrane ruffles. When subjected to a scratch wound assay, cells depleted of Rab5C, but not Rab5A or Rab5B, demonstrated reduced cell migration. U937 cells depleted of Rab5C also displayed reduced cell motility in a Transwell plate migration assay. To examine activation of Rac, HeLa cells stably expressing GFP-Rac1 were independently depleted of Rab5A, Rab5B or Rab5C and seeded onto coverslips imprinted with a crossbow pattern. 3-D GFP-Rac1 images of micro-patterned cells show that GFP-Rac1 was less localized to the cell periphery in the absence of Rab5C. To confirm the connection between Rab5C and Rac activation, HeLa cells depleted of Rab5 isoforms were starved and then stimulated with EGF. Rac1 pull-down assays revealed that EGF-stimulated Rac1 activity was significantly suppressed in Rab5C-suppressed cells. To determine whether events upstream of Rac activation were affected by Rab5C, we observed that EGF-stimulated Akt phosphorylation was suppressed in cells depleted of Rab5C. Finally, since spatio-temporal assembly/disassembly of adhesion complexes are essential components of cell migration, we examined the effect of Rab5 isoform depletion on the formation of focal adhesion complexes. Rab5C-depleted HeLa cells have significantly fewer focal adhesion foci, in accordance with the lack of persistent lamellipodial protrusions and reduced directional migration. We conclude that Rab5 isoforms selectively oversee the multiple signaling and trafficking events associated with the endocytic network.

## Introduction

Rab5, the prototypical Rab GTPase identified [1] and localized [2] almost 25 years ago, operates as a master regulator of the endocytic pathway [3]. Rab5 regulates homotypic endosome fusion [4], [5] molecular motor-driven vesicle movement on microtubules [6] and Rab conversion [7], the process by which Rab GTPases along a transport pathway are kept in register. Rab5 also plays a central role in the internalization and trafficking of signal transducing cell surface receptors [8].

Rab5 is encoded as three isoforms, Rab5A, Rab5B and Rab5C in mouse and human genomes. These isoforms are encoded by different genes and expressed in all tissues [9]. Bucci et al. [10], [11] examined Rab5 isoform function in cultured cells and showed that expression of all three Rab5 isoforms independently affect endocytosis. Subsequently, Rab5 isoforms were found to be differentially phosphorylated, suggesting that they serve as more than a backup or redundant function in endocytosis [10]. More recent work has extended the idea that the Rab5 isoforms have different, if overlapping, functions. Wainszelbaum et al. [12] and Bhattacharya et al. [13] reported that Rab5 isoforms are differentially induced by cytokines. Chen et al. [14] recently reported that Rab5A is selectively coupled with EGFR degradation and that Rin1, a guanine nucleotide exchange (GEF) factor, shows specificity towards Rab5A activation. Moreover, in contrast to Rab5A, silencing of Rab5C had no effect on EGFR trafficking.

The complex and diverse role of Rab5 isoforms in endocytic transport is highlighted by the large number of proteins with which they interact. Rab5 is activated by as many as six GEFs including Rabex5 [15], Gapex-5 [16], Rin1 [17], Rin2 [18], [19], Rin3 [20] and Als2 [21] and deactivated by at least two Rab5 GTPase-activating proteins (GAP) RabGap-5 [22] and RN-Tre [23]. Rab5 effectors include EEA1 [24] and Rabinosyn-5 [25], proteins that mediate Rab tethering to membranes through the well-characterized FYVE domain [26], and APPL1 and APPL2 [27], proteins that interact with Rab5 to orchestrate membrane trafficking and that affect gene transcription. PI3 kinase [28] and PI5 and PI4 phosphatases [29] interact with Rab5 to regulate aspects of signal transduction and the temporal regulation of phosphoinositide turnover required for progression of cargo through the early endocytic pathway. This collection of Rab5 isoform effectors, GEFs and GAPs form a large interactive network that orchestrates and regulates the multiple functions associated with the early endosomal compartment.

The current study builds on our earlier work on Rab5 isoform specificity [8], [14] and on the work of Palamidessi el.al, who showed that Rab5 and the Rab5 GAP, RN-Tre, modulates Rac activity and cell motility [30], [31]. Here, we examined the differential effects of Rab5 isoform silencing on cell motility. We report that Rab5C, but neither Rab5A nor Rab5B, is selectively associated with the growth factor-activation of Rac1 and with enhanced cell motility.

## Materials and Methods

### Antibodies

Monoclonal anti-Rab5A antibody, polyclonal anti-Rab5B and anti-Rab5C antibodies used in this study have been described previously [14]. Monoclonal anti-Rac1 (cat# 610650) was from BD transduction laboratories. Mouse anti-PIP3 and FITC-PIP3 antibodies were purchased from Echelon Biosciences Inc. (Salt Lake City, UT, USA) and rabbit anti-p110 and monoclonal anti-GFP antibodies from Santa Cruz Biotechnology (CA). p-FAK(Y397) antibody are purchased from Cell signaling Technology.

### Plasmids

cDNA of Rab5A, Rab5B and Rab5C were subcloned into SalI/ BamHI sites of pEGFP-C1 (Clontech). GST-PAK1-CRIB construct was generously provided by Dr. A. Barbieri (Florida International University, Miami, Florida, USA).

### pSUPER RNAi constructs and stable cell line

Sequences targeting individual Rab5 isoforms were adapted from oligo siRNA as described previously [14] and cloned into pSUPER-neo-GFP vectors (OligoEngine) at *Bgl*II /*Hind*III sites. The oligo primers for hRab5A are:

5′-GATCCCCGAGTCCGCTGTTGGCAAATTTCAAGAGAATTTGCCAACAGCGG ACTCTTTTTA and 5′-AGCTTAAAAAGAG TCCGCTGTTGGCAAATTCTCTTGAA ATTTGCCAACAGCGGACTCGGG; for hRab5B are: 5′-GATCCCCAAGACAGCTA TGAACGTGATTCAAGAGATCACGTTCATAGCTGTCTTTTTTTA and 5′-AGCTT AAAAAAAGACAGCTATGAACGAGATCTCTTGAATCACGTTCATAGCTGTCTTGGG; for hRab5C are: 5′-AGCTTAAAAAAATGAACGTGAACGAAATCTCTCTTG AAGATTTCGTTCACGTTCATTGGG and 5′-GATCCCCAAT GAACGTGAACGAA ATCTTCAAGAGAGATTTCGTTCACGTTCATTTTTTTA

Cloning of pSUPER RNAi constructs was carried out according to the instructions. HeLa cells were transfected with indicated pSUPER RNAi constructs with Lipofectamine™ 2000 (Invitrogen). Cells were selected with neomycin (1–2 mg/ml) for 3–4 weeks. Several clones were isolated and tested for Rab5 isoform down-regulation

### siRNA construction and transfection

The siRNAs against Rab5 isoforms were constructed and purified using the Silencer™ siRNA construction kit (Ambion, Austin, TX) as previously described [14]. A scrambled siRNA (Ambion) or siRNA designed against GFP was used as negative controls. The transfection of siRNA (20 nM final concentration) was performed using Lipofectamine™ 2000 (Invitrogen) following the manufacturer’s instruction.

### Cell culture and Analysis

HeLa cells were maintained in Dulbecco’s modified Eagle’s medium (Invitrogen) supplemented with 10% bovine growth serum (Hyclone Laboratories) containing penicillin and streptomycin. Stable HeLa-GFP-Rac1 cells are kind gifts from Dr. W. D. Hardt (ETH Zuerich). Statistical analysis was carried out using one-way and two way analysis of variance (ANOVA) with Dunnett’s and Bonferroni’s post-test.

### Immunoblotting and Immuno-precipitation

As described earlier [14] cell lysates were prepared with lysis buffer containing protease inhibitor cocktail (Sigma). The cell lysates were clarified by centrifugation prior to separation by SDS-PAGE. The resolved proteins were transferred to nitrocellulose membranes (Whatman Schleicher & Schuell, Florham Park, NJ) and then blocked in TBST containing 5% nonfat milk. The membranes were probed with primary antibodies followed by HRP-conjugated secondary antibodies (Jackson ImmunoResearch, West Grove, PA). Proteins were visualized by enhanced chemiluminescence detection reagents (Pierce). Immunoblot data were quantified by AlphaEaseFC 4.0 software (Alpha Innotech Corp. San Leandro, CA). Immuno-precipitations were carried out with clarified cell lysates, incubated with primary antibodies and protein G-Sepharose (Sigma) overnight at 4°C. The beads were washed extensively with lysis buffer and solubilized in SDS sample loading buffer.

### Scratch wound assay

HeLa cells were plated on a 3.5 cm glass-bottom dish (Fisher Scientific) the day before siRNA transfection. 48 hours after transfection, several 0.5–1 mm width wounds were made across the confluent cell monolayer using a standard 200 µl pipette tip. The wounded monolayer was washed twice to remove non-adherent cells and then incubated in fresh medium. Multiple (5–7) microscopic fields were observed in each culture dish with the 10X objective of an inverted, wide-field video microscope (Leica DMIRE2, Leica Microsystems, Wetzlar, Germany) connected to a CCD camera and a computer. Phase-contrast images from each selected wound area were taken every 5 minutes for 20 hours. The time-lapse images collected from each wound were processed with ImageJ to generate movies.The wound edges were outlined and the wound areas were calculated with ImageJ from photos taken at time 0 and 16 hours. Percentage of wound closure was calculated as (Wound area t = 16h -Wound area t = 0)/Wound area t = 0). The graph represents Mean±SE of four independent experiments each with five different wound closure images [10].

### Rac1 activation

Rac1 activation was assayed using the p21-binding domain (PBD) of PAK fused to glutathione S-transferase (GST) [32]. Immediately after EGF stimulation (100 ng/ml), cells were lysed in Rac1 lysis buffer (50 mM Tris (pH 7.5), 10 mM MgCl_2_, 200 mM NaCl, 1% Nonidet P-40, 5% glycerol), and 1 mg of total lysate was incubated with GST-PBD beads at 4°C for 1 h. Beads were collected by centrifugation and were washed three times with washing buffer (25 mM Tris -HCl (pH 7.6), 30 mM MgCl_2_, 40 mM NaCl, 1% Nonidet P-40). Proteins were eluted by boiling beads in SDS sample buffer, separated on a 12% SDS-PAGE, and blotted for Rac1.

### Transwell migration assay

U937 cells were transfected with siRNAs using Nucleofector II (Amaxa Biosystems). 48 hours post-transfection, cells were rinsed once and resuspended in serum-free medium. 2×10^5^ of U937 cells were seeded in the transwell insert (Falcon 3μm FluorBlock 24-well Inserts) placed in 24-well cell culture insert companion plate. Medium with 10% FCS is added to the bottom well to stimulate cell migration. Images of migrated cells in the bottom chamber were taken with inverted light microscope after 24 hours. Numbers of cells were counted using Image J “Analyze Particle”. At least 5 fields of cell images were taken per treatment. Migration was evaluated as percent of cells migrated over initial cell number.

### Focal adhesion complex formation and FAK activation assay

HeLa cells were seeded on coverslips overnight and then transfected with siRNA against Rab5 isoforms or GFP (as control). 48 hours after transfection, cells were fixed in 4% paraformaldehyde, permeablized and stained with vinculin antibody to visualize focal adhesion complexes. Confocal images were taken using MRC1024 (Bio-Rad Laboratories) microscope with 63X objective. The numbers of focal adhesion complexes were determined using the “analyze particle” function in ImageJ. For the FAK activation study, HeLa cells were transfected as indicated above. 48 hour post-transfection, cells were re-suspended and re-plated on fibronectin-coated plates for the indicated times. At each time point, cells were rinsed with ice-cold PBS, proteins were extracted with lysis buffer and prepared for SDS-PAGE and immunoblotting. The activation of focal adhesion kinase was determined with phospho-FAK antibody.

### Micropatterned Cell Imaging

12 mm glass coverslips were imprinted with crossbow micropattern following method developed by Azioune et al. 2009 (Azioune, A., Storch, M., Bornens, M., Thery, M. and Piel, M. (2009) Simple and rapid process for single cell micro-patterning. *Lab Chip*, 9, 1640–1642.). Coverslips were coated with poly(L-lysine)-*graft*-poly(ethylene glycol) (PEG-g-PLL) and then subjected to UV irradiation under a crossbow pattern chrome photomask. Next, the micropatterned coverslips were coated with fibronectin. Cells transfected with indicated siRNAs were seeded onto the micropatterned coverslips. For GFP-Rac1 localization, HeLa cells stably expressing GFP-Rac1 were spread out for 2-3 hours on the micropatterned coverslips and then fixed with 4% PFA. For detection of PIP3 production, cells were seeded on micropatterned coverslips in serum-free medium for 2–3 hours, and then were stimulated with 20% FCS for 3 minutes. Immediately after stimulation, cells were fixed, permeablized and then stained with FITC-PIP3 antibody. 3D image stacks of GFP-Rac1 or FITC-PIP3 staining were acquired using DeltaVision deconvolution microscope. Defined slices from each image stack were subjected to max intensity projection. In each experiment, at least 30–40 cells were imaged for every treatment (control or knock down (KD)). The projected images (n = 30–40) from the same treatment were made into a stack, aligned and then averaged using Image J (average intensity projection). The average intensity projections from different samples were normalized to obtain equal maximum and minimum grey value. To determine the differences of GFP-Rac1 localization, projected average intensity of Rab5 KD samples were subtracted from that of control. The resulting subtraction images represent the localization of intensity differences in cells between control and KD samples. 3 independent experiments were carried out with similar results.

### Cell fractionation

Hela cells were washed, scraped and harvested in the homogenization buffer (10 mM Tris/acetic acid, pH 7, 250 mM sucrose). The cell pellet was resuspended with the same buffer and homogenized by 15 passes through a 25 gauge needle. The cell homogenate was centrifuged at 4000 g for 10 min to pellet cell debris and nuclei. The supernatant was centrifuged at 100,000 g for 20 min to separate cytosol and cell membranes. The membrane pellet was resuspended in homogenization buffer with 1% TX-100 for 30 minutes at 4°C. Samples were centrifuged again at 12000 g for 10 minutes to pellet the insoluble proteins. Equal volume of cytosol and membrane fractions were used to analyze GFP-Rac1 enrichment.

## Results

### Loss of Rab5C alters cell shape and dampens cell motility

**Figure 1:**
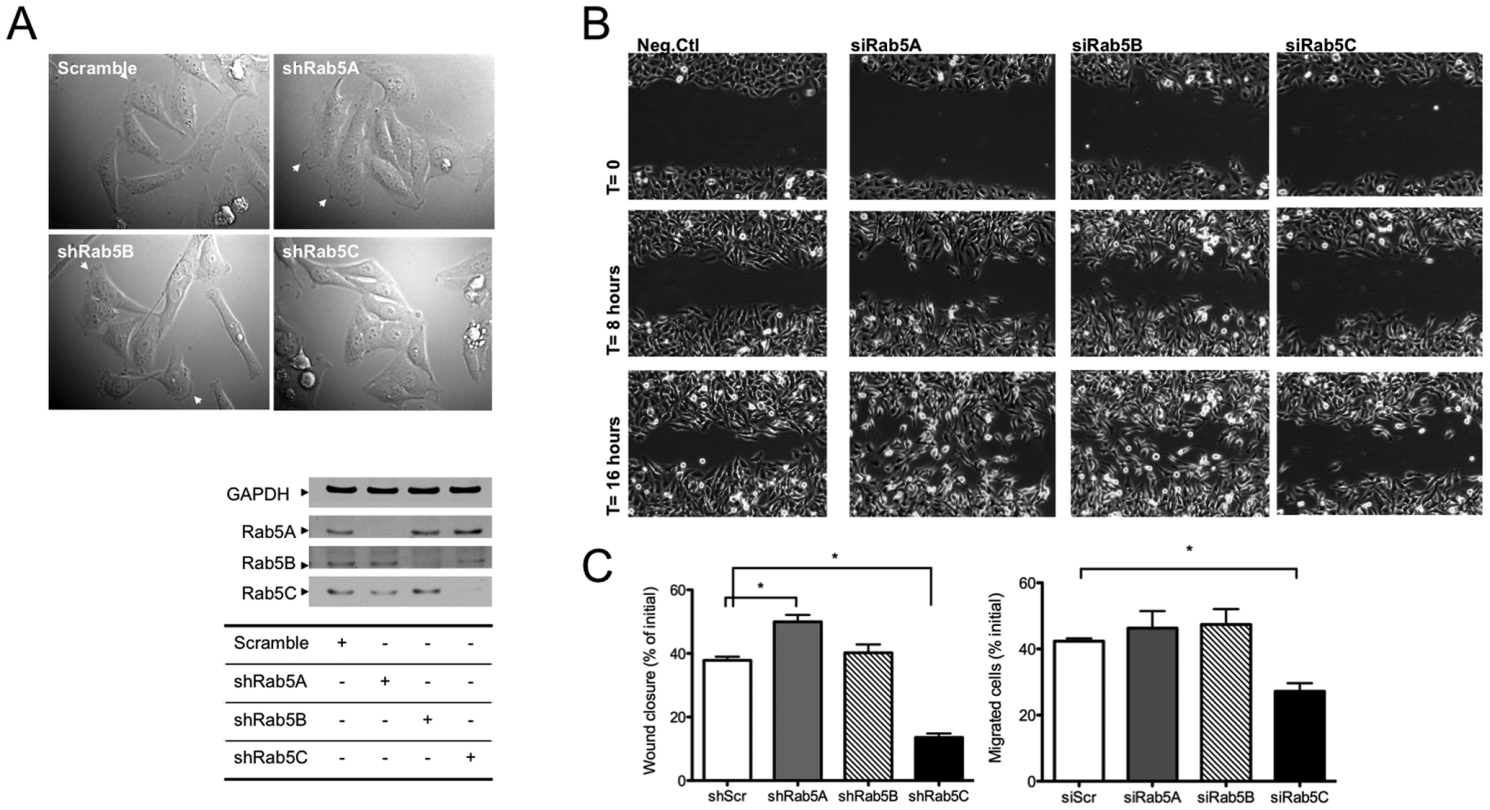
Rab5C depletion significantly inhibits cell migration. A) DIC images of stable Rab5 isoform knock-down (KD) HeLa cells taken with light microscope at 40X magnification (left panel). Arrows indicate membrane ruffles. KD of Rab5 isoforms (right panel) in these stable cell lines is shown in the immunoblots following SDS-PAGE as described in Experimental Procedures. B) 0.5–1 mm width wounds were made on a monolayer of HeLa stable control or Rab5 isoform KD cells. 5–7 wounded spots in each dish were imaged with time-lapse microscope every 5 minutes for 20 hours. C) The percentage of wound closure (left panel) was calculated from images acquired at time 0 and 16 hours with ImageJ. For each sample, at least 5 images were used to calculate the percentage of wound closure in each experiment. The graph represents the Mean± S.E. from four independent experiments. U937 cells (right panel) transiently transfected with siRNA against Rab5 isoforms were seeded in the upper chamber of the Transwell plates and allowed to migrate towards 10% FBS in the bottom chamber for 24 hours. Migrated cells were measured as indicated in Material and Methods. Data are normalized to initial seeding cell numbers. The graph represents the Mean± S.E. from four independent experiments. Analysis was carried out with a one-way ANOVA, Dunnett’s post-test.(*P<0.05, ***P<0.001)

To investigate cellular functions specific for each Rab5 isoform, we established HeLa cell lines stably depleted of individual Rab5 isoforms using pSUPER vector system (OligoEngine). Figure 1A (right panel) shows the level of individual Rab5 isoforms following silencing. We found that control cells and cells stably depleted of Rab5A or Rab5B had characteristic HeLa cell morphology with well-defined membrane protrusions. Rab5C silencing, however, led to spindle-shaped cells and reduced formation of membrane ruffles (Figure 1A (left panel)). We speculated that the morphological change in Rab5C KD cells would impair their ability to migrate. To test this hypothesis, scratch wound assays were carried out to evaluate the migration rate of these stable KD (knock-down) cells (Figure 1B). Time-lapse video microscopy showed that in the absence of Rab5C, cells were significantly less motile and the majority of the outermost cells oriented parallel to the scratch wound during migration. As opposed to Rab5C depletion, Rab5A or Rab5B-silenced cells quickly became perpendicular to the wound and developed persistent lamellipodia with high directionality and velocity. Quantification of the wound closure data indicates that Rab5C depletion strongly inhibits cell migration, while the loss of Rab5A mildly enhanced it (Figure 1C, left panel). To verify that cell migration is differentially regulated by Rab5 isoforms, U937 cells were transfected with siRNA against Rab5 isoforms and examined for trans-migration towards a serum gradient. The data in Figure 1C (right panel) show that Rab5C depletion (see Figure S1) significantly suppresses U937 migration whereas silencing of either Rab5A or Rab5B had no effect.

### Silencing of individual Rab5 isoforms leads to differential Rac1 activation

**Figure 2:**
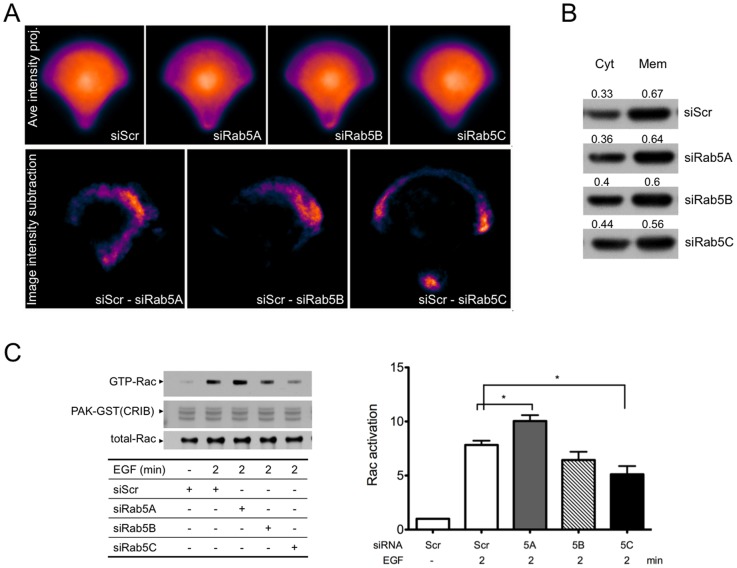
Loss of Rab5C reduced translocation of Rac1 to cell periphery and Rac1 activation in response to EGF stimulation. A) HeLa cells stably expressing GFP-Rac1 were transfected with scrambled or Rab5 isoform siRNAs. 48 hours post-transfection, cells were seeded onto coverslips imprinted with crossbow micro-patterns. 3-D GFP-Rac1 images of at least 40 micro-patterned cells were acquired for each sample. Each GFP-Rac1 3-D image stack was subjected to Maximum intensity projection and then grey scale normalization. Next, the max projections of 88 GFP-Rac1 images (from two independent experiments) were aligned and averaged (upper panel, in pseudo-color). Image subtraction was carried out between averaged image of scramble control and that of individual Rab5 isoform siRNA-treated sample. The resulting image after subtraction (siScr minus siRab5) is shown in pseudo-color (bottom panel). B) HeLa cells stably expressing GFP-Rac1 were transfected with scrambled or Rab5 isoform siRNAs. 48 hours post-transfection, cells were separated into membrane (Mem) and cytosolic (Cyt) fractions as described in Material and Method. Relative amounts of GFP-Rac1 in each fraction were analyzed by SDS-PAGE and Western blot. Densitometry of the bands was quantified using AlphaEaseFC 4.0 software. The numbers represent the ratio of GFP-Rac1 in cytosol or membrane/total. C) HeLa cells were transfected with scrambled or Rab5 isoform-specific siRNA. 48 hours post-transfection, cells were starved and then stimulated with EGF for two minutes. Cell lysates were prepared and subjected to Rac1 pull-down assays. Proteins were eluted, separated by SDS-PAGE and blotted for Rac. Total lysates were also probed for Rac1 to determine the total Rac1 level is equal in all samples. The intensity of the bands from western blots was quantified with AlphaEaseFc 4.0 software. The relative amount of Rac-GTP from pull-downs was normalized to that of total Rac1 from total cell lysates. The adjacent graph represents the mean value ± S.E. from four independent experiments. Analysis was carried out with a one-way ANOVA, Dunnett’s post-test. (*P<0.05, **P<0.01)

Rac1 is a critical regulator of membrane ruffle formation and cell migration. Rac1 is activated at the plasma membrane and promotes lamellipodium extension in response to motogenic stimuli. Palamidessi el al. [31] showed that expression of Rab5 enhanced Rac1 activation on endosomes and transformed stationary cells to adopt motile morphology. It is possible that the different effects of Rab5 isoform KD on cell migration were due to differential regulation of Rac1 membrane association and/or activity. To test this hypothesis, HeLa cells stably expressing GFP-Rac1 were seeded on fibronectin-coated crossbow micro-patterns that allowed cells to take the shape that mimics migration. The localization of GFP-Rac1 was imaged with a 3D deconvolution microscope. For each image stack, several image slices closest to the substrate were projected to visualize membrane-associated GFP-Rac1. The average intensity projection derived from several tens of maximum intensity projected cell images provided an overall intensity distribution of the GFP-Rac1 in control and KD cells. We then subtracted the GFP-Rac1 image intensity of KD cells from that of control cells. Figure 2A (top panel) shows the average intensity projection of GFP-Rac1 in control scrambled cells and in cells depleted of Rab5A, Rab5B or Rab5C. Figure 2A (bottom panel) represents the differences of GFP-Rac1 intensity between control and KD cells. These data show that GFP-Rac1 was less localized to the cell periphery in the absence of Rab5C. To further demonstrate that the membrane association of GFP-Rac1 was perturbed by Rab5C depletion, control or Rab5 isoform KD cells were fractionated to separate cytosolic and membrane bound GFP-Rac1. Indeed, we found that Rab5C depletion moderately reduced the association of GFP-Rac1 with the membrane fraction (Figure 2B). To examine Rac1 activation, GST-PAK1-CRIB (Cdc42/Rac1 interactive binding domain) pull-down assays were carried out to quantify the levels of GTP-bound Rac1 in cells stimulated with EGF. As shown in Figure 2C (left panel), EGF-stimulated Rac1 activity was significantly suppressed (Figure 2C (right panel) in Rab5C KD cells. On the contrary, Rab5A KD appears to modestly enhance Rac1 activation. This modest but reproducible elevation of Rac1 activity could be due to prolonged EGFR signaling, as we have previously shown that Rab5A KD delays EGFR degradation [14]. Rescue experiments with Rab5C KD stable cells and RNAi resistant constructs of the Rab5 isoforms confirmed that Rab5C was much more effective in restoring Rac activation by EGF than Rab5A (see Figure S2).

### Rab5C KD reduced PI3-kinase activity

**Figure 3:**
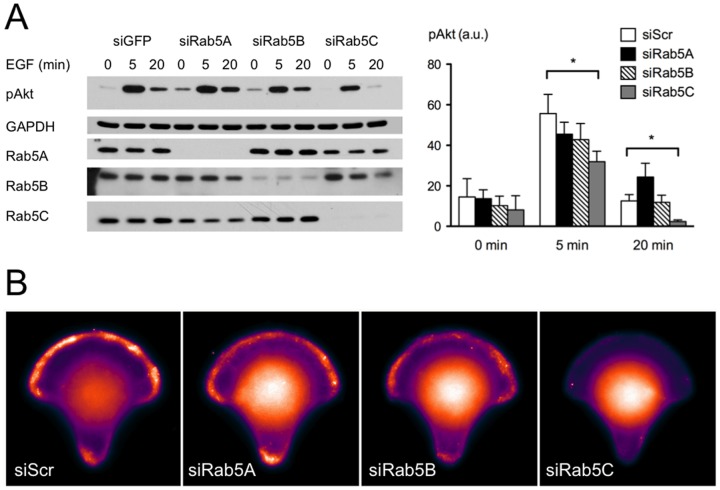
PI3K signaling in response to Rab5 isoform depletion. A) HeLa cells were transfected with GFP (as negative control) or Rab5 isoform-specific siRNA. 48 hours post-transfection, cells were starved and then stimulated with EGF for indicated times. Cell lysates were subjected to SDS-PAGE and probed with antibodies as indicated. Band intensity was quantified with AlphaEaseFc 4.0 software. Bars represent the mean value ± S.E. from four independent experiments. Analysis was carried out with a two-way ANOVA, Bonferroni’s post-test. P<0.05. B) HeLa cells were transfected with indicated siRNA. 48 hours post-transfection, cells were seeded onto micropatterned coverslips coated with fibronectin, and then allowed to spread out for 2 hours in starvation medium. Starved cells were stimulated with 10 % FCS for 3 minutes and then fixed for PIP_3_-FITC antibody immuno-staining. Images shown here are average projections of PIP_3_ staining from 30–35 cells.

EGF-stimulated Rac1 activation is rapid and transient. Both PI3 kinase and Ras play key regulatory roles in this process. A large body of work shows that PI3K activates Rac1 via PtdIns(3,4,5)P_3_-sensitive Rac-GEFs, such as Vav, SOS1 and Tiam1; whereas Ras enhances Rac1 activation through both PI3K-dependent and -independent pathways. To better understand the mechanism that caused the inhibition of Rac1 activation in the absence of Rab5C, we tested how PI3K activity responded to Rab5 isoform depletion. We found that silencing Rab5C significantly inhibited EGF-stimulated Akt phosphorylation (Figure 3A). Moreover, experiments with stable Rab5 knock down cells showed that EGF stimulation was much less able to stimulate Rac and akt activation in Rab5C knock down cells (Figure S3).

As Akt/PKB is one of the proximal downstream targets of PI3K, the suppression of its phosphorylation suggested that PI3K signaling is altered or suppressed. Meanwhile, immunostaining of KD and control cells on the crossbow micro-pattern with PIP_3_-specific antibody also showed a substantial loss of PIP3 signal on the plasma membrane when Rab5C was silenced (Figure 3B). Collectively, these data indicate that Rab5C regulates, directly or indirectly, PI3 kinase activity, thereby Rac1 activation and cell migration is preferentially inhibited by Rab5C depletion.

### Rab5C KD reduced cell adhesion

**Figure 4:**
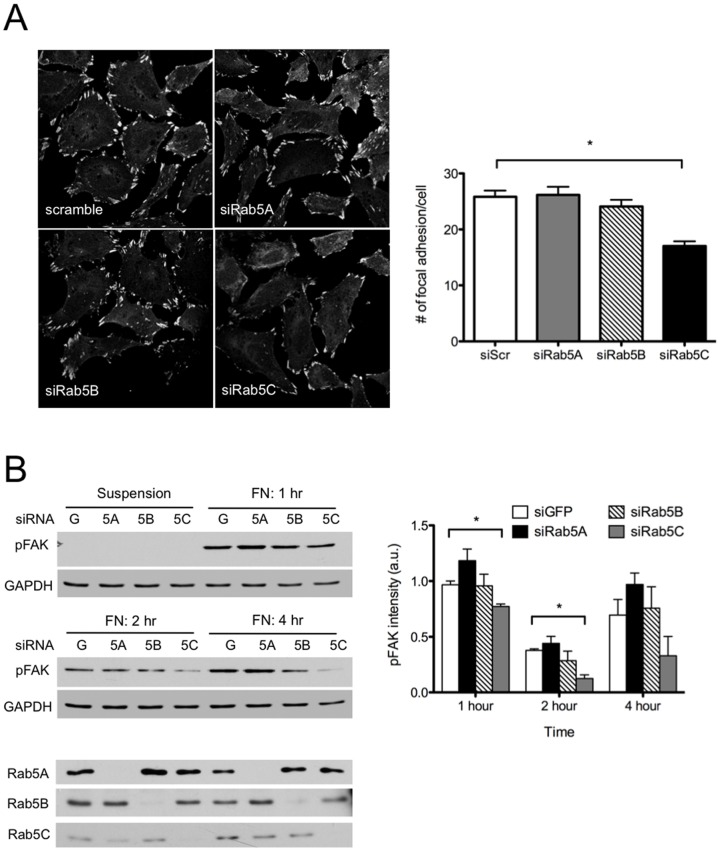
Depletion of Rab5C reduces cell adhesion. A) HeLa cells were seeded on coverslips O/N and then transfected with GFP or Rab5 isoform siRNAs. The focal adhesion complex was visualized by immunostaining with vinculin antibody. The numbers of focal adhesion complexes were determined with ImageJ. The graph represents Mean± S.E. from 30 cells. Analysis was carried out with a one-way ANOVA, Dunnett’s post-test. P<0.0001. B) HeLa cells transfected with GFP or Rab5 isoform siRNAs were re-suspended and re-plated on fibronectin-coated plates for indicated times. At the end of each time point, cell lysates were extracted and prepared for SDS-PAGE and Western bloting. The activation of focal adhesion kinase was determined with phospho-FAK antibody. Total levels of FAK were not determined. The data represents Mean± S.E. from three independent experiments. Analysis was carried out with a two-way ANOVA, Bonferroni’s post-test. P<0.05.

Cell migration is a multi-step process involving cell polarization, cell membrane protrusion at the leading edge, and spatio-temporal assembly/disassembly of adhesion complexes. To further delineate the differential migratory behaviors of Rab5 isoform-silenced cells, we also examined the formation of focal adhesion complexes. As shown in Figure 4A, Rab5C-depleted HeLa cells have significantly fewer focal adhesion foci, which is in accordance with the lack of persistent lamellipodial protrusions (Figure 1A) and reduced directional migration.

Focal adhesions are highly dynamic structures that form at sites of membrane contact with the extracellular matrix. During migration, focal adhesion kinase (FAK) coordinates the assembly/disassembly of focal adhesions via its phosphorylation and subsequent recruitment of focal adhesion complex-associated proteins. In the absence of Rab5C, cells re-plated onto fibronectin substrates had dampened FAK activation over time (Figure 4B), suggesting that the dynamics of cell adhesion is impaired in these cells.

## Discussion

The current study builds on earlier work showing that Rab5A selectively regulates growth factor receptor trafficking [8], [14] and focuses on the role of Rab5C in selectively regulating cell motility and cytoskeletal dynamics. DiFiore and colleagues have suggested that Rab5 acts as a critical switch in the endocytic circuitry by which Rac1 can be activated and re-routed to specific sites at the plasma membrane to initiate actin assembly [31]. More recently, the same group has demonstrated that the Rab5 GAP RN-Tre, delays the turnover of focal adhesions clearly indicating a role for Rab5 in cell migration. In their study, Rab5 was examined by silencing all three Rab5 isoforms [30]. Here we show that Rab5C preferentially serves this function via modulating a combination of signaling and trafficking pathways.

The correlation of Rac1 activation with Rab5 isoform silencing/over-expression was tested both at steady state (data not shown) and under EGF stimulation. We found that all three Rab5 isoforms were capable of potentiating Rac1 activity following exogenous expression (Figure S4). On the contrary, Rac1 activation responded to the individual depletion of endogenous Rab5 isoforms very differently. Loss of Rab5C function suppressed Rac1 activity both at steady state and when stimulated by EGF. Rab5B depletion showed only mild suppression. In correspondence with the reduced Rac1 activity, Rab5C-depleted cells exhibited altered cell shape and defective locomotion towards open wound space in a scratch-wound assay or in a trans-well migration assay with a serum gradient (Figure 1C and Supplemental Figure 1). These findings suggest that Rab5C plays a preferential role in Rac1-mediated cell migration. In contrast to over-expression, depletion of Rab5A mildly increased Rac1 activity and cell motility. The discrepancy between RNAi depletion and over-expression of Rab5A is likely the sum of motogenic signaling pathways and endocytic events. We reasoned that while overexpression of Rab5A, as well as other isoforms, can elevate endocytosis and enhance Rac1 activation, Rab5A KD delays EGFR degradation and prolongs its signaling [14]. Consequentially, KD of Rab5A may increase Ras-GTP levels that potentially mediate Rac1 activation through both PI3K-dependent and –independent mechanisms. Activated Ras can initiate a positive feedback loop by direct interaction with p110 [33], thereby increasing PtdIns(3,4,5)P_3_ levels at the leading edge. Activated PI3K may further enhance Ras activation through PtdIns(3,4,5)P_3_-mediated stimulation of Gab1 phosphorylation and recruitment of Grb2/SOS. The PI3K-independent mechanism involves interaction of Ras-GTP and Tiam1, which subsequently activates Rac [34]. For these reasons, we believe Rab5A-depleted cells have overall more stimulatory motogenic signals. Since Rab5C does not appear to regulate EGFR degradation, its loss of function migratory response is not skewed by the EGFR-Ras-Rac1 activation cascade.

We explored the possibility that endogenous Rab5C shows more specificity towards Rac-induced cell migration via the PI3K pathway. Rab5 not only interacts with both catalytic (p110β) and regulatory subunits (p85α) of PI3 kinase, but enhances the PI3K activity. It is unclear if the interaction between PI3K and Rab5 is isoform-specific, but the inhibition of pAkt and PIP_3_ production in response to Rab5C depletion does suggest that Rab5C preferentially modulates PI3K activity. One other possibility that could explain the differential effects on cell motility in response to individual Rab5 isoform depletion is an unbalanced endocytic trafficking of membrane adhesion proteins, such as cadherins and integrins. Cadherins that are internalized by several routes pass through Rab5- and EEA1-positive early endosomes, and the cell’s adhesive potential depends upon whether the adhesion molecules are sorted to lysosomes for degradation or recycled back to the cell surface [35]. In zebrafish, prechordal plate progenitor cells exhibit active migratory behavior toward the animal pole of the gastrula using the overlying ectoderm as a substrate on which to migrate. E-cadherin is required for prechordal plate progenitor spreading at the interface between mesoderm and ectoderm and subsequent migration during later stages of gastrulation. Recently, the dynamics of E-cadherin turnover at the plasma membrane was found to be modulated by Rab5C-mediated endocytosis due to its sole expression at this developmental stage [36]. Consistent with these findings, our data showed that Rab5C depletion significantly reduces the formation of cell focal adhesion, and the activity of focal adhesion kinase. A similar finding was recently reported by Mendoza et al. [37] although Rab5C was not specifically tested. Though the current study did not address whether Rab5 isoforms differentially regulate the trafficking of adhesion molecules, recent reports indicate that Rab5C operates semi-independently from the other Rab5 isoforms by interacting directly and apparently selectively with AMAP1 [38], thereby linking Rab5C to a growth factor-stimulated integrin recycling pathway that regulates cell invasion.

The mechanism that controls the specificity of Rab5 isoform function may rely on the location where the isoform is most prominently activated thereby selectively affecting endosomal sorting and signaling events. Rab5A interacts more favorably with the Rab5 GEF, Rin1 when cells are stimulated with EGF [14]. Rin1 interacts with the EGF- and other growth factor receptors through its SH2 domain thereby linking Rab5A activation with EGFR signaling. As mentioned above, Rab5C selectively interacts with AMAP suggesting that Rab5C may be recruited to the Arf6/integrin pathway. It is also possible that Rab5C interacts with the exocyst, the macromolecular complex that regulates the activation of Rac1 and cell motility [39], [40]. A recent paper by Blumer and colleagues [41] proposes that the interaction between Rabs and their exchange factors along with other Rab interacting proteins is responsible for controlling the targeting of individual Rabs to their appropriate localization. With the range of Rab5 GEFs currently cataloged and other Rab tethering factors such as EEA1, this could be the most attractive explanation for the selective targeting of the Rab5 isoforms to endosomal sub-compartments.

Interestingly, Stenmark and colleagues [42] reported many years ago that EEA1 preferentially interacts with Rab5B in a yeast two-hybrid screen suggesting that the activation of the various isoforms of Rab5 are variable (i.e., that they may be differentially controlled by the collection of GEFs and possibly GAPS).

In summary, silencing of individual Rab5 isoforms showed distinct biological responses – suppression of Rab5A delays growth factor receptor trafficking while silencing of Rab5C suppresses Rac1 activation, cell shape, membrane ruffle formation and PI3K activity. We suggest that the Rab5 family evolved, along with any number of Rab5 effectors and activators, to orchestrate a “division of labor” to accommodate a more complicated endocytic pathway found in vertebrates.

## Supporting Information

Figure S1 **KD of Rab5 isoforms in U937 cells.** U937 cells were transfected with 20 nM of siRNA against Rab5 isoforms or scrambled siRNA using Nucleofector II (Amaxa Biosystems). 48 hours post-transfection, cells were centrifuged, washed with PBS and lysed in Lysis buffer. Cell lysates were run on SDS-PAGE, and KD of each Rab5 isoforms were determined with indicated antibodies.
(TIF)

Figure S2 **Rab5 isoform expression restores Rab5CKD suppressed Rac1 activation.** HeLa cells were co-transfected with scrambled or Rab5C siRNAs along with GFP, GFP-Rab5A, 5B or 5C (RNAi-resistant) constructs using Lipofectamine 2000. 48 hours post-transfection, cells were starved for 4 hours and then stimulated with EGF (100 ng/ml). Cell lysates were subjected to PAK1-GST pull down to determine GTP-bound Rac1. The level of Rac activation is presented as GTP-Rac/total Rac in the adjacent graph.
(TIF)

Figure S3 **Stable Rab5C KD suppresses Rac activity.** HeLa cells, stably knocked down of Rab5 isoforms with scrambled or Rab5C shRNAs, were starved for 4 hours and then stimulated with EGF (100 ng/ml) for 2 minutes. Cell lysates were subjected to PAK1-GST pull down to determine GTP-bound Rac1. The level of Rac activation is presented as GTP-Rac/total Rac in the adjacent graph.
(TIF)

Figure S4 **Rab5 isoform expression enhances Rac activation.** HeLa cells were transfected with CFP alone or CFP-Rab5 isoforms. The Rac-GTP was measured by p21-binding domain pull down (PD) assay following EGF stimulation as indicated.
(TIF)
