# Supplementary figures and images for: Rab5 Isoforms Orchestrate a “Division of Labor” in the Endocytic Network; Rab5C Modulates Rac-Mediated Cell Motility
Source: PLoS One. 2014 Feb 28;9(2):e90384. doi: 10.1371/journal.pone.0090384 (PMC3938722; doi:10.1371/journal.pone.0090384)

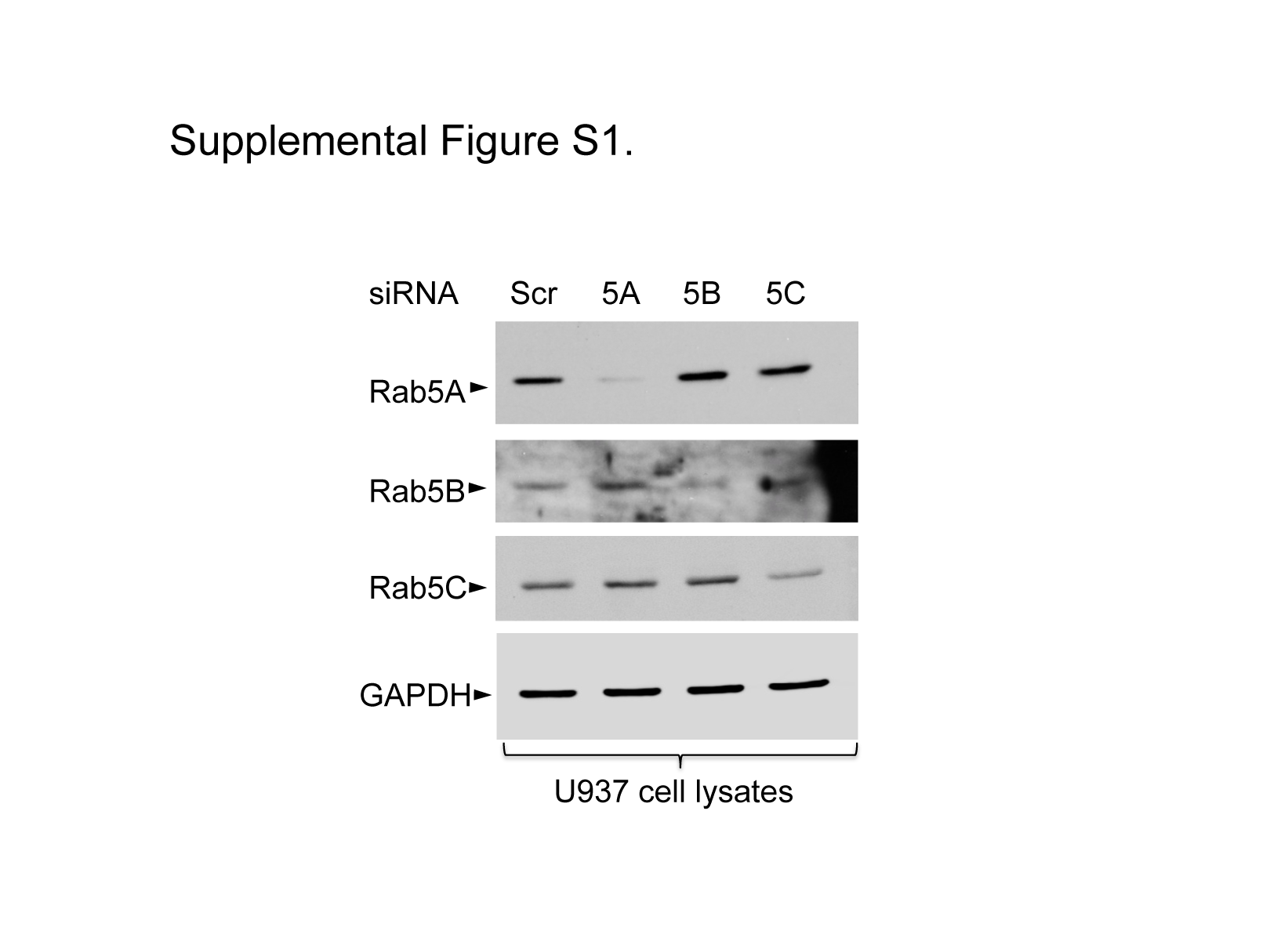

Supplement: Figure S1 — KD of Rab5 isoforms in U937 cells. U937 cells were transfected with 20 nM of siRNA against Rab5 isoforms or scrambled siRNA using Nucleofector II (Amaxa Biosystems). 48 hours post-transfection, cells were centrifuged, washed with PBS and lysed in Lysis buffer. Cell lysates were run on SDS-PAGE, and KD of each Rab5 isoforms were determined with indicated antibodies. (TIF) [file pone.0090384.s001.tif]

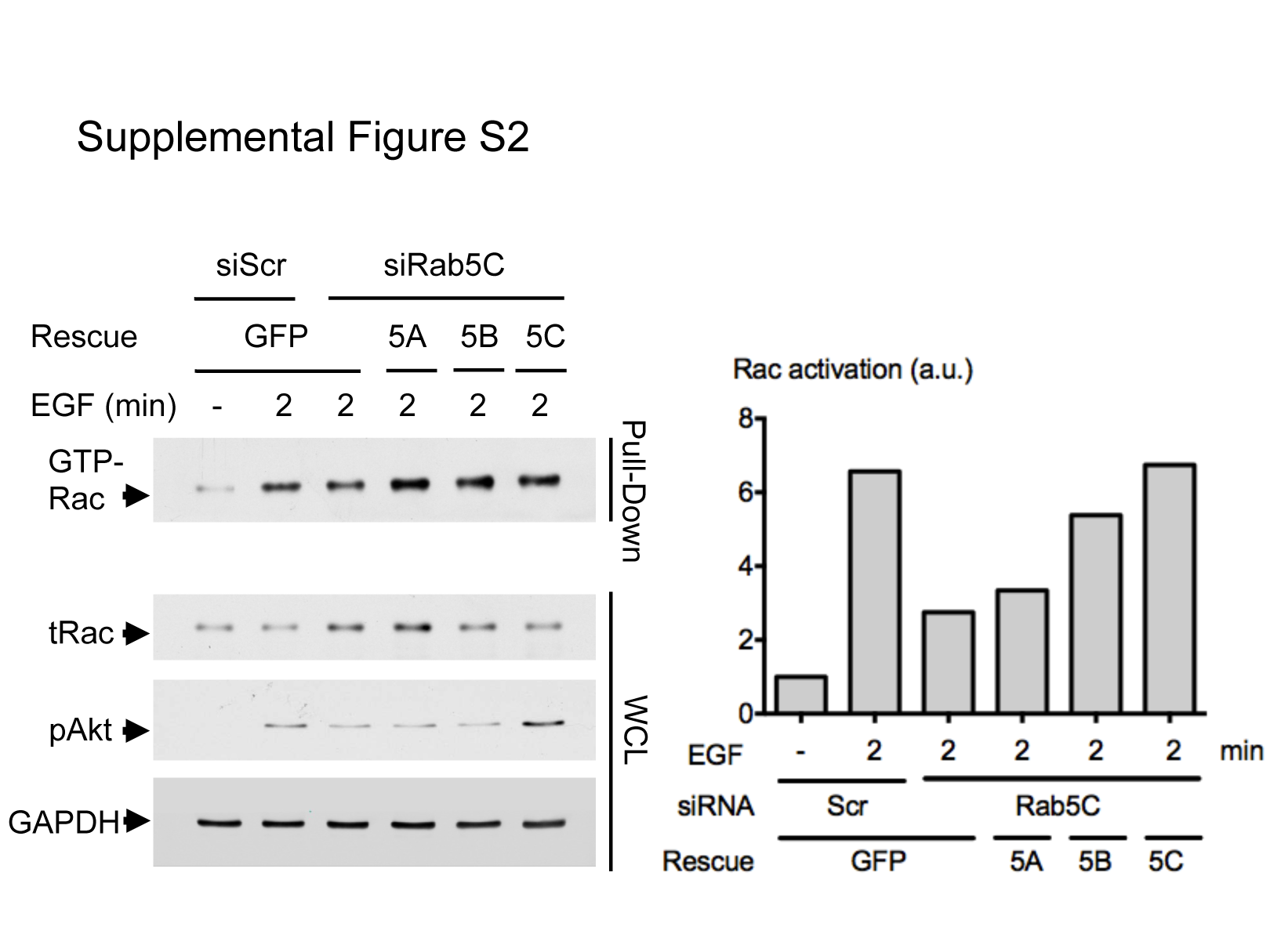

Supplement: Figure S2 — Rab5 isoform expression restores Rab5CKD suppressed Rac1 activation. HeLa cells were co-transfected with scrambled or Rab5C siRNAs along with GFP, GFP-Rab5A, 5B or 5C (RNAi-resistant) constructs using Lipofectamine 2000. 48 hours post-transfection, cells were starved for 4 hours and then stimulated with EGF (100 ng/ml). Cell lysates were subjected to PAK1-GST pull down to determine GTP-bound Rac1. The level of Rac activation is presented as GTP-Rac/total Rac in the adjacent graph. (TIF) [file pone.0090384.s002.tif]

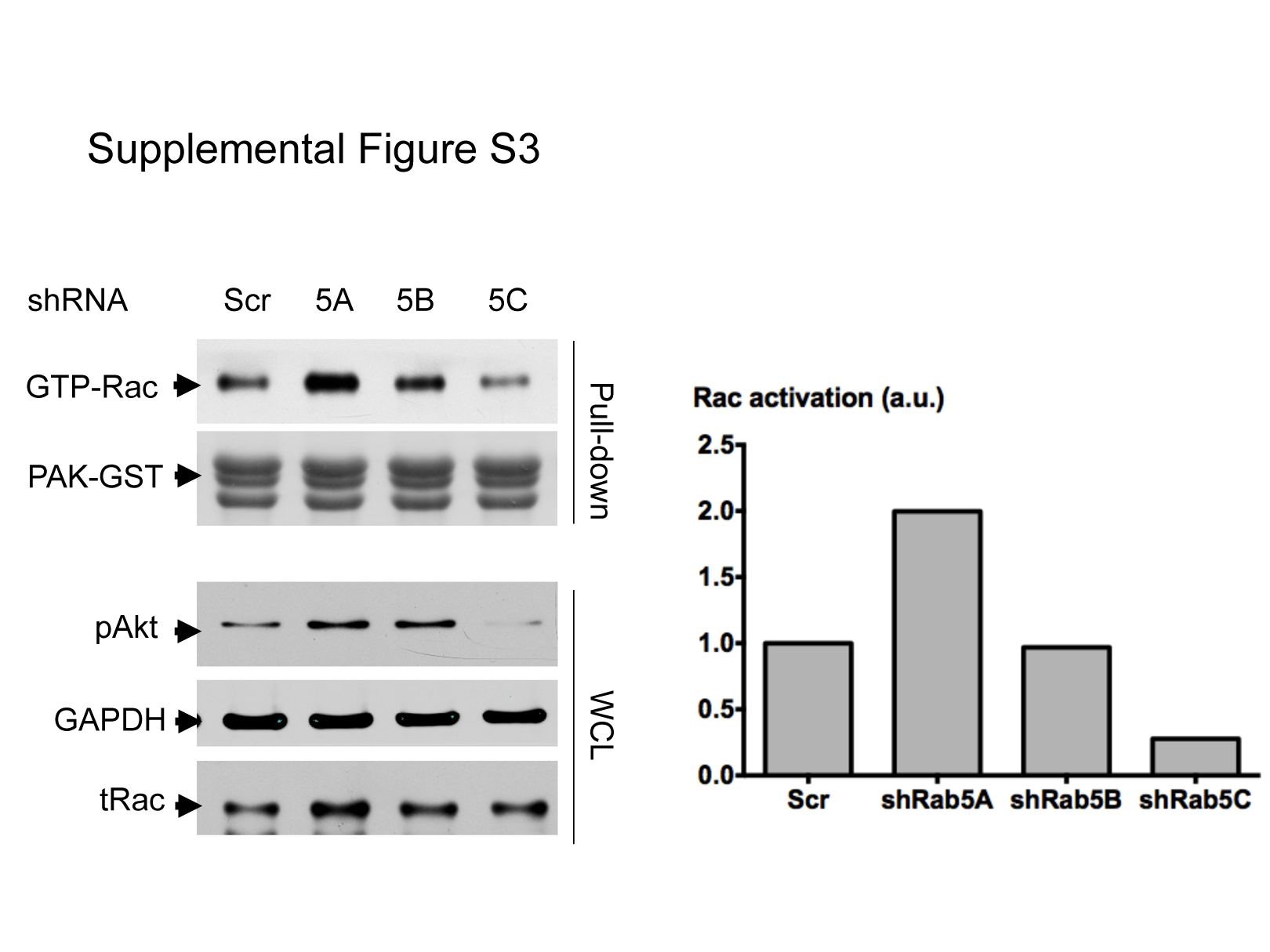

Supplement: Figure S3 — Stable Rab5C KD suppresses Rac activity. HeLa cells, stably knocked down of Rab5 isoforms with scrambled or Rab5C shRNAs, were starved for 4 hours and then stimulated with EGF (100 ng/ml) for 2 minutes. Cell lysates were subjected to PAK1-GST pull down to determine GTP-bound Rac1. The level of Rac activation is presented as GTP-Rac/total Rac in the adjacent graph. (TIF) [file pone.0090384.s003.tif]

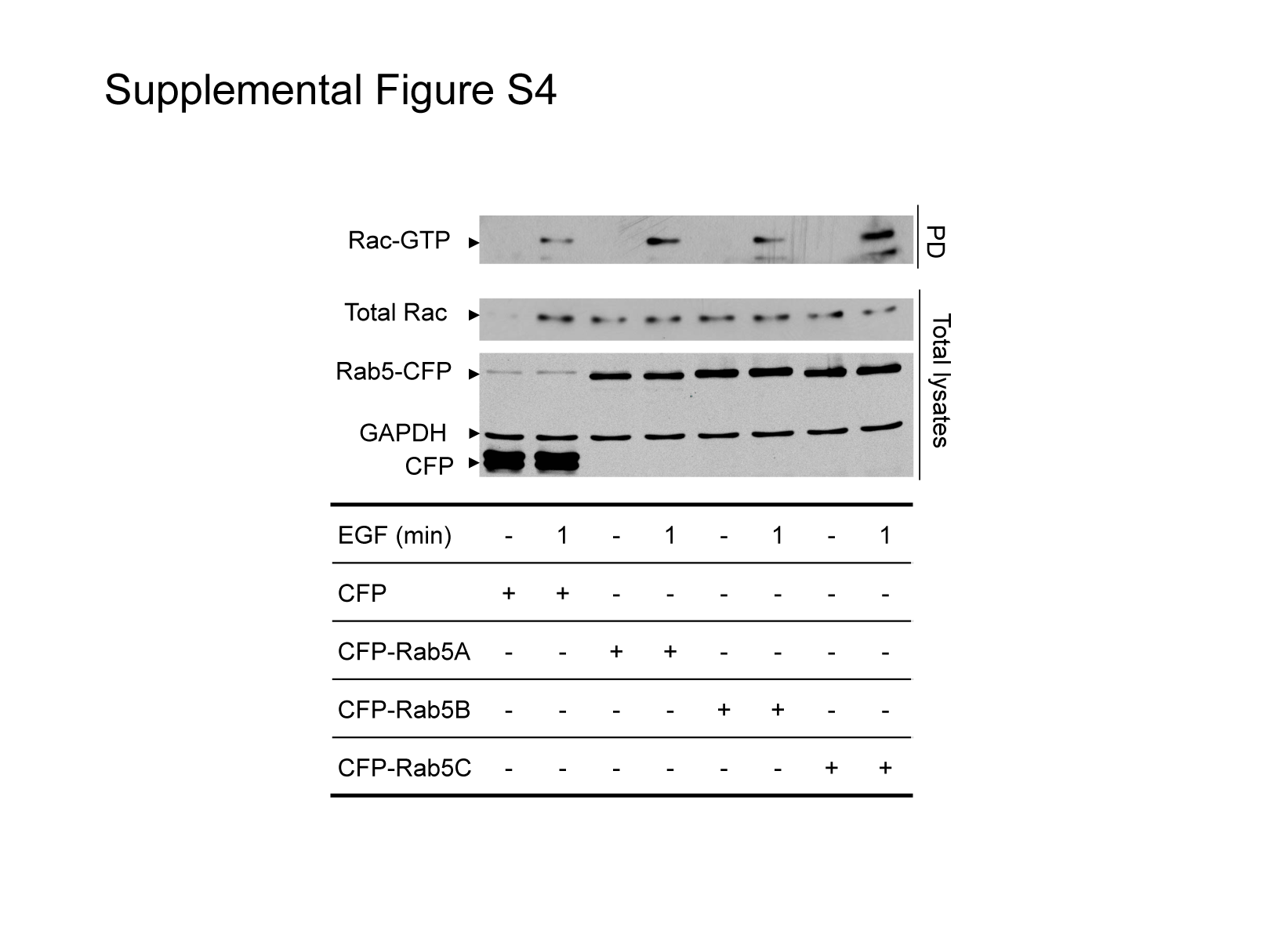

Supplement: Figure S4 — Rab5 isoform expression enhances Rac activation. HeLa cells were transfected with CFP alone or CFP-Rab5 isoforms. The Rac-GTP was measured by p21-binding domain pull down (PD) assay following EGF stimulation as indicated. (TIF) [file pone.0090384.s004.tif]
